# Case of Placenta Praevia, Treated by Prof. Simpson’s Method

**Published:** 1845-12-01

**Authors:** I. Jones


*Case of Placenta Praevia treated by Prof. Simpson's method, by I. Jones, Esq.* We find the following in the New York Medical Intelligencer, quoted from the London Lancet. We should be glad to have a report of the adoption of this method by some of our friends, should any meet with a case of placenta praevia favorable for its application.

"I was summoned, on the 14th of March, to a poor woman in labor with her sixth child, who was represented as having profuse hæmorrhage, which had continued almost uninterruptedly for about four hours. On my arrival, I found my patient in a most precarious state from exhaustion, with a pulse averaging from 130 to 140; she was almost speechless, slightly delirious, and there was no uterine action. There was then no hæmorrhage. I was informed she had had repeated slight attacks of hæmorrhage for six weeks previously. Having rallied her with twenty drops of tincture of opium combined with weak brandy and water, I proceeded to make an internal examination. Before I could exactly ascertain the state of the parts, I was deluged with a frightful gush of blood. The os uteri was fully dilated, the membranes entire, the presentation of the head natural; and the placenta covered nearly two-thirds of the os uteri. The hæmorrhage persisting, and there being now considerable uterine pains, I saw no time was to be lost. I accordingly, and without the least difficulty, insinuated my fingers between the adherent portion of the placenta and the uterus, and in less than a minute, the latter was freed of the placenta. I now administered a full dose of the tincture of ergot, which brought on strong expulsive pains, and the woman was delivered of a still-born child in one hour and thirty-five minutes after my entering the house. After the placenta had been extracted, I do not think she lost altogether, more than a teacupful of blood; she recovered as soon as females generally do after an ordinary labor, excepting that she continued unusually weak for a considerable time, as was to be expected after the great loss of blood. She had one similar labor before; the child was then turned with great difficulty (her pelvis not being capacious,) peritonitis ensued, and she very narrowly escaped with her life, after a confinement to her bed of more than two months."

